# The courage to venture: Revealing the effect of social trust on corporate venture capital

**DOI:** 10.1371/journal.pone.0295844

**Published:** 2023-12-22

**Authors:** Yue Zhang, Xiaoxu Guo

**Affiliations:** College of Finance, Nanjing Agricultural University, Nanjing, People’s Republic of China; Government College University Faisalabad, PAKISTAN

## Abstract

In an uncertain and risky business environment, the decision for corporate venture capital (CVC) often requires courage and determination. This article empirically examines the relationship between social trust and corporate venture capital based on CVC data from Chinese companies spanning from 2006 to 2018. The findings reveal that social trust significantly positively influences a company’s willingness and scale of involvement in venture capital. Further analysis highlights the variations in social trust effects under diverse governance environments, particularly in non-state-owned firms and firms with separate CEO and chairman roles. Meanwhile, in regions characterized by a more developed market environment and a robust legal framework, social trust demonstrates a more pronounced motivating effect. Moreover, social trust fosters innovation within CVC deals. Focused on emerging markets, this research delves into the significance of informal institutions in incentivizing corporate innovation and venture capital, offering a fresh perspective on the driving forces behind CVC.

## 1 Introduction

Trust is a particular level of the subjective probability with which an agent assesses that another agent or group of agents will perform a particular action [1], and is an essential manifestation of social capital [2,3]. Over the past 20 years, the word “trust” has received much attention in the field of economics and finance [3–6]. Economists tend to define social trust as: A lubricant for the construction and functioning of the social economy, which is not only a fundamental element of transactional behavior [7] but also a public good necessary for the economy [8]. At the macro-level, social trust could contribute to economic prosperity [3], drive economic growth [9,10], develop financial markets [11], and improve laws and government regulations [4]. When involving the micro-level, social trust could offer trade credit [12], enhance the level of risk-taking [13], reduce the probability of corporate misconduct [14], influence corporate environmental strategic decisions [15], encourage corporate financing, cross-border mergers and acquisitions [16,17]. Many economists have intuitively recognized the significance of trust in economic transactions, suggesting that social trust contributes to building credibility in contractual relationships. Social trust comes to the fore when the scope of transactions is limited, and economic agents deprive each other due to misconduct. The literature is less clear, however, about how social trust affects venture capital at the firm level.

This paper examines the influence of social trust on corporate venture capital (CVC) decision-making by addressing the following three key questions: Does a high level of social trust impact a company’s decision to engage in corporate venturing, thereby promoting active CVC initiatives? What are the situational factors that modulate the relationship between social trust and CVC decision-making? How does increased social trust enhance the effectiveness of CVC investments in companies already engaged in CVC activities?

The article provides predictions in three aspects surrounding these three issues. Firstly, the probability of established companies engaging in venture capital is positively correlated with the level of trust in the region where the business operates. Secondly, under different organizational characteristics and environmental contexts, the role of social trust in incentivizing companies to choose venture capital investments varies. Thirdly, the paper attempts to make some predictions regarding the additional benefits brought by CVC in the context of social trust.

To test the predictions, the article uses a hand-collected dataset of Chinese CVC activities from CVSource from 2006 to 2018. The rich dataset enables this research to encompass a variety of company-specific characteristics, through which social trust can influence investment and outcomes. The CEI index is used to measure social trust, derived from the “China Urban Commercial Credit Environment Index”. Measures of CVC participation include the number of CVC deals and the investment size of CVC. The data clearly show that social trust has a positive impact on CVC. The article specifically proposes that social trust can stimulate CVC through two channels. First, social trust reduces transaction costs caused by information asymmetry and decreases the friction in information during transactions. Second, social trust contributes to providing stable psychological expectations for participants and creates a culture that tolerates failure, thereby reducing managers’ concerns when making decisions.

Additionally, to explore the underlying mechanisms, this paper further examines the mediating roles of firm and regional characteristics. The conclusion shows that various internal and external factors influence the functioning of social trust as an informal institution. The positive effect of social trust on CVC is more salient for firms with non-state-owned status and CEO duality, also good market and legal environment promote the effect of social trust. Furthermore, consideration is given to the influence of social trust on CVC returns from both financial and strategic perspectives. Evidence suggests that social trust enhances the value creation capability of CVC, as reflected in greater corporate innovation output.

The article empirically studies the link between social trust and CVC based on data from China, since China provides an interesting and ideal backdrop. First, there are disparities in social trust in the locations of Chinese listed companies. Different degrees of taxation, legislation, economic policies and corporate governance offer a natural experimental environment for the consequences of social trust [12,18]. Second, China is in a transitional period where investor protection is still inadequate and enforcement is relatively weak [17]. The potential function of social trust may be more prominent in emerging market setting with less transparency and less stringent information disclosures [19]. Therefore, this paper can conduct a stronger test of the relation between social trust and CVC.

This paper builds on and contributes to, a number of literature. Ang et al. [17] and Fonseka et al. [20] document that higher social trust is associated with more investment. Bottazzi et al. [21] use the Eurobarometer to measure trust between countries and find a positive relationship between trust and venture capital. This study provides complementary yet distinct analyses. The main attention is drawn to the behavior of venture capital made by companies rather than investing institutions. Most relevant studies of CVC focus on markets in developed countries, and there is little recent empirical research on corporate venture capital organizations related to developing countries. The sample of China during the transition period is utilized to contribute to the study of economic development in emerging markets.

Compared with the existing literature, the contribution of this paper contains the following main aspects. First, this study adds research on the micro perspective under the positive influence of social trust. Prior research has examined the impact of social trust on economic growth and financial market development [6], but firm-level evidence is limited. The role of social trust in the most fundamental activity of firms—investment behavior, remains relatively unexplored. Using a sample of Chinese firms in the emerging market, our work contributes to the body of knowledge regarding how social trust affects corporate behavior.

The second contribution is that this study expands the study of the influencing factors of CVC. Focusing on CVC as a specific type of venture capital, this paper explores the impact of informal institutions on CVC decisions. The cognition on the drivers of CVC remains extremely limited [22], with minor literature focused on macro-environmental level effects. Scholars mainly concentrate on the influence of micro-enterprise-level factors on CVC, such as executive characteristics [23], firm performance [24], social ties [25], technology-related factors and demand conditions [26], and geographical location [27]. Based on the external environmental factors, the analysis delves into the driving mechanism of CVC in China from the perspective of social trust and effectively combines the macro trust environment with the economic decision of micro enterprises, which has important practical significance for promoting corporate venture capital and stimulating corporate innovation.

The third contribution is providing a comprehensive portrayal of the disparate effect of social trust under different internal and external governance environments. Based on the Chinese stage of economic development and the extent of social trust, this research examines the moderating effects of state ownership, CEO duality, marketization level and legal environment, which enrich and expand the literature on the mechanisms of social trust in the micro-firm level under Chinese institutional scenarios. Discussing the vulnerability and validity of social trust under the changes in internal and external factors can provide implications for the correct perception of the current influence of social trust. The facts prove that the influence of social trust is closely related to the internal and external governance environment.

The fourth contribution is that the article validates the return on CVC from social trust, exploring the effects on financial performance and innovation output respectively. It turns out that the existence of social trust is the primary condition for CVC to reap a better return on investment. This paper provides theoretical implications for creating a great trust environment, enhancing inter-firm contracts and trust, and amplifying investment effects.

The remainder of the paper proceeds as follows. Section “Hypotheses Development” discusses the prior literature and develops research hypotheses. Section “Sample Selection and Empirical Design” includes data collection and variable measurement. Section “Empirical Results” reports the empirical results. Lastly, conclusions, discussions and limitations are provided in Section “Conclusion and Discussion”.

## 2 Hypotheses development

### 2.1 Social trust and corporate venture capital

Social trust is an essential integrated social force [28], which maintains the efficient operation of economic activities and affects the scale of transactions [3]. In practically all economic activity, it serves a crucial enabling function [29]. Once trust is absent, so many things cannot be done. CVC is a corporate economic activity and inherently a transaction between companies [30]. Investing in start-ups is a risky venture. Despite its potentially high returns [31], the investment process is fraught with uncertainty and risk. Start-ups lack sound financial information, credit records and hard assets in the early stages of establishment, which makes it difficult to assess the business value and actual operating conditions. Due to great concerns about the real development and growth prospects of start-ups, investors often make decisions after weighing strategic goals, financial returns and risk management [32].

How does social trust work in CVC transactions? According to the economic theory of information, information asymmetry is common since different transaction subjects have varied capacities for knowledge acquisition and information access [33]. Extensive literature in economics and sociology shows that trust reduces the transaction costs of information asymmetry in all exchange relationships with potential opportunism risks [34–37]. The hypotheses of this article are based on these findings. Similar to venture capital, CVC transactions involve deal origination, screening, evaluation, deal structuring and post-investment activities [38,39], and each link faces great uncertainty. In a situation of information asymmetry, it is hard for investors to have an active willingness to take risks. Social trust increases access to information and raises the quality of the available information [40], which essentially prevents the spread of moral hazards and opportunistic behavior in investment transactions [41]. When the trading relationship is maintained by social trust, the reliance on contracts between the trading parties decreases. There is less conflict and uncertainty in the transaction process [42], reducing the cost consumed at each link. Thus, as a kind of social capital, creates great external conditions for efficient venture capital. Social trust influences the CVC through information sharing and decision mechanism, helping to sign and execute contracts between the parties of transactions.

The function of social trust in the trading process is to provide participants with stable psychological expectations [43] and enhance individual self-motivation [41]. As a lubricant and fulcrum for cooperation [29,44], social trust could effectively strengthen corporate capabilities of risk-taking [13,45], and encourage firms to engage in venturing [21,46]. Trust is a psychological implication for oneself that the trade counterparty is trustworthy, and it is frequently used to explain a firm’s choice to interact and associate with other firms [47]. This paper considers that in regions with higher levels of social trust, corporate managers are more likely to trust others and initiate external investment. Trust increases the investor’s desire to take risks through positive psychological cues. Investors are willing to place faith in the information provided by the target party and actively engage in communication and contact. On the other hand, when investors are from areas with high moral standards, business trustworthiness is also generally high. With good coordination between investors and investees, it is easier for start-ups to recognize and accept external investment from these regions. Therefore, hypotheses are proposed as follows.

**H1a:** In the region with higher social trust, companies are more likely to conduct venture capital.
**H1b:** In the region with higher social trust, companies tend to invest more in venture capital.

### 2.2 Heterogeneity analysis

This article explores the mechanisms of social trust on CVC from internal and external governance environments respectively, in order to investigate the consequences of social trust in various circumstances.

#### 2.2.1 The role of state ownership between social trust and CVC

Social trust offers investment firms external possibilities to engage in venture capital, but the capacity to seize and profit from such opportunities differs. The state ownership of firms determines the internal institutional environment in which managers make investment decisions. CVC deals generally invest in start-ups, with a long investment cycle, high uncertainty and high opportunity cost [24]. State-owned enterprises tend to be cautious and pursue conservative investments in the face of uncertainty [48]. Meanwhile, state-owned enterprises with relatively stable governance structures have a tendency to adopt less risky activities [49]. These firms are often subject to a certain degree of intervention by government and regulatory agencies [50], and their business decisions are widely scrutinized. State-owned enterprises need to go through layers of reporting and auditing when investing. The specific destination and use of funds are often strictly verified, which is a cumbersome process and may result in higher transaction costs.

Moreover, most state-owned enterprise managers have administrative backgrounds. In the face of performance evaluation and position promotion, their willingness to avoid innovation risks still exists even if motivated by a trusting atmosphere. Executives of non-state-owned enterprises are generally adventurous and possess a higher willingness to take risks. They are eager to enhance corporate technical innovation through CVC to reduce marginal costs, improve product quality, maintain sustainable competitive advantage and maximize corporate value. This leads to the following hypothesis.

**H2a:** Compared with state-owned enterprises, the promotion effect of social trust on CVC is more pronounced in non-state-owned enterprises.

#### 2.2.2 The role of CEO duality between social trust and CVC

CEO Duality is the situation when a CEO, besides running the corporation at the highest level, also holds the position of the chairman of the board. CEO duality affects firm performance, value and business behavior [51], and influences CVC decisions [23]. The combination of CEO and chairman of the board gives the CEO enormous power [52], which leads to the lack of necessary supervision and control in the process of corporate business decision-making. Managers may focus on immediate financial performance rather than potential long-term strategic gains [53]. When the CEO and chairman of the board are not the same person, the right of control is separated from ownership [54], preventing the opportunistic behavior of management. Separating the duties of CEO and chairman can improve governance and information handling in conditions of greater environmental uncertainty, particularly with regard to strategic decisions or disruptive technology choices [55,56]. The following hypothesis is therefore posited.

**H2b:** The separation of CEO and chairman promotes the positive effect of social trust on CVC activity.

#### 2.2.3 The role of marketization level between social trust and CVC

Social trust may be affected by other external environmental elements [57], such as economic and social factors. The level of marketization serves as a comprehensive indicator of both economic and social aspects [58]. The relationship between social trust and CVC varies for firms at different stages of marketization. In the region with higher marketization, firms’ external social resource allocation will be more efficient. Higher information transparency results from a good market environment [59], which gives investors access to adequate market information, investment opportunities and better climate for growth. Therefore, the improvement of external market mechanism shows a mutually reinforcing effect on social trust. Based on this, this paper proposes the following hypothesis.

**H2c:** A higher degree of marketization increases the contribution of social trust to CVC.

#### 2.2.4 The role of legal environment between social trust and CVC

Douglas C. North, the Nobel laureate in economics, defines institutions as the rules of the game in society, the constraints determining how people relate to each other. New institutional economics divides institutions into formal institutions (such as regulations and laws) and informal institutions (social ethics, values, moral norms, etc.). Formal institutions, informal institutions and the joint implementation of both could affect and constrain the transaction of economic markets [60]. When formal institutions fail to protect investors’ interests, investors increasingly rely on natural trust in economic decision-making [61]. At this time, social trust, an informal institution, is crucial in regulating trading behavior and facilitating transactions. For countries in transition economies where formal institutions are not yet sound, informal institutions largely ensure the smooth signing and performance of transaction contracts [3,62,63]. Based on the empirical research in Italy, Guiso et al. [11] also prove that informal institutions perform better in financial development in areas where law enforcement is inefficient. In this way, social trust compensates for the weakness of formal institutions to a certain extent. Thus, the following hypothesis is stated.

**H2d:** In regions with weaker legal systems, social trust as an informal institution positively promotes CVC.

### 2.3 Social trust and CVC effect

The effectiveness of the investment is greatly influenced by the level of information asymmetry between the investor and the investee. Early in the investing process, disagreements and conflicts may take some time for both parties to settle down. When divisions arise between investors and entrepreneurs, strong trust helps a lot to solve problems [64]. In a region with higher social trust, the investors could trust the investee to a greater degree and have the courage to take risks. Investors believe that start-ups have the will and ability to achieve rapid growth. Start-ups are more likely to recognize these inward investments from high-trust areas, thinking that the investors can perform their management functions and boost the development of the start-ups. Both parties can engage in equal communication, information sharing and collaborative work toward worthy goals based on trust.

The reciprocal positive relationship between social trust and investment success has been confirmed [65]. Further, higher social trust contributes to more corporate investment and payoffs [21]. Established companies initiate CVC deals for strategic or financial goals, acquiring new technologies or exploring new business developments. When a company makes an investment decision, CVC links the investor and the investee into a tightly knit whole. Social trust is a key factor that affects the performance and return of the investing enterprise, especially in the link of knowledge transfer [66,67]. Compared with VC, established companies conducting CVC are more accurate in knowing the advantages and potential problems of new ventures.

The integration of complementary resources is more easily achieved in a context of social trust. Established companies optimize and share the value contributed by start-ups through contributing their knowledge and talents, leading to promising investment returns [68,69]. Social trust is also a powerful mechanism for maintaining the CVC contract during the resource integration process. Social trust helps keep deals implemented smoothly and correct deviations promptly, which drives both parties to achieve better cooperation [70]. Consequently, social trust is a favorable condition in the transaction [29], which promotes CVC activities with positive effects. Thus, our testable hypothesis is as follows.

**H3** In regions with higher social trust, CVC deals can deliver superior effects.

## 3 Sample selection and empirical design

### 3.1 Sample selection

Our initial sample consists of all Chinese A-share listed firms covered by the *China Stock Market and Accounting Research* (CSMAR) database from 2006 to 2018. Financial companies and companies with missing data of relevant indicators are then excluded. After applying these data filters, 27,823 company-year observations are obtained to conduct the empirical tests.

### 3.2 Variable definition

#### 3.2.1 Measure of social trust

Social trust is an informal institution formed by society and building a stable social value. In this paper, social trust is measured by the China Urban Commercial Credit Environment Index (CEI), which is compiled by the China Academy of Management Science. The index is collected from the report of *China City Commercial Credit Environment Index*, which provides the score of social trust among China’s provinces. This index ranges from 0–100, with a higher score indicating a better business trust environment in the region. The index ranges from 0–100, with a higher score indicating a better business trust environment in the region. CEI has been widely used in recent sociological and economic studies [71,72]. In this study, TRUST is the natural logarithm of social trust and subjective beliefs for the location of investors. Scores from a questionnaire-based *entrepreneurial survey system* are also used as a measure of social trust in the robustness test.

#### 3.2.2 Measure of CVC

CVC is whether a company has carried out CVC deal in the year [25,73]. The CVSource database records every CVC investment of Chinese listed company. The value of CVC is 1 if the company has implemented a CVC deal; otherwise, 0. The second measure of CVC is AMOUNT which represents the size of CVC [74], collecting the sum of venture capital of a company for the year.

#### 3.2.3 Moderator variable

The moderating variable contains four indicators, including state ownership (SOE), CEO duality (DUAL), marketization level (MARKET) and legal environment (LAW). If the enterprise is state-owned, SOE is assigned a value of 1; otherwise, 0. If the CEO and chairman are the same person, the value of DUAL is 1; otherwise, 0. The data of SOE and DUAL is obtained from the CSMAR database. The level of marketization is used to measure the degree of marketization of the economy in which the enterprise is located. The legal environment represents the formal institutional environment in which the firm is located. The LAW and MARKET indices are in the range of 0–10, from *Marketization Index of China’s Provinces*: *NERI Report 2018* [58]. MARKET and LAW are divided by average of the year, assigned 1 and 0 respectively.

#### 3.2.4 Control variable

Following the previous studies [20,22,24], control variables in the analysis included governance, financial and organizational features. The data all comes from the CSMAR database. First, governance-related characteristics are considered, including ownership concentration (TOP1), the ratio of independent director (IDR) and holding of venture capital (VC). TOP1 is the percentage of shares held by the company’s largest shareholder. IDR is measured by the number of independent directors as a percentage of the total board members. If the established company has an infusion of VC funds, it means a higher degree of adventure spirit and a natural preference for venture capital [27]. VC is equal to 1 if the established company is supported by private venture capital funds; otherwise, 0. Second, financial characteristics are considered, containing ROA, LEV and GROW. ROA is the ratio of net profit divided by total assets, representing the profitability of the company. LEV is the ratio of total liabilities to total assets, representing the ability to repay debts. GROW is measured by the growth rate of revenue from core business. Third, organizational properties, including SIZE and AGE are factored into the analysis. SIZE is the natural logarithm of corporate assets. Age is the natural logarithm of the company’s establishment time. Finally, year and industry are also fixed to control for the possible impact of time trends and industry differences on CVC.

### 3.3 Empirical models

There are two units of observation. In the first part of the analysis, the focus is on the investment decision, that is, whether a firm engaging in a CVC deal. To this end, a sample of all potential deals is constructed, using model (1) to estimate the probability of a firm instituting a CVC deal. CVC is a binary variable in the model (1). Additionally, AMOUNT is set as a dependent variable to measure the size of CVC in the model (2).


$$CVC=\beta_{0}+\beta_{1}TRUST+\beta_{2}TOP1+\beta_{3}IDR+\beta_{4}VC+\beta_{5}ROA+\beta_{6}LEV+\beta_{7}GROW+\beta_{8}SIZE+\beta_{9}AGE+\beta_{10}SOE+\sum Y\mathrm{ear}+\sum I\mathrm{ndustry}+\varepsilon$$ (1)

$$AMOUNT=\beta_{0}+\beta_{1}TRUST+\beta_{2}TOP1+\beta_{3}IDR+\beta_{4}VC+\beta_{5}ROA+\beta_{6}LEV+\beta_{7}GROW+\beta_{8}SIZE+\beta_{9}AGE+\beta_{10}SOE+\sum Y\mathrm{ear}+\sum I\mathrm{ndustry}+\varepsilon$$ (2)

In the second part, the research focus is on the effects brought by CVC under different levels of social trust. To this end, model (3) is used to estimate the CVC effect influenced by social trust.


$$EFFECT=\beta_{0}+\beta_{1}TRUST+\beta_{2}TOP1+\beta_{3}IDR+\beta_{4}VC+\beta_{5}ROA+\beta_{6}LEV+\beta_{7}GROW+\beta_{8}SIZE+\beta_{9}AGE+\beta_{10}SOE+\sum Y\mathrm{ear}+\sum I\mathrm{ndustry}+\varepsilon$$ (3)

## 4 Empirical results

### 4.1 Descriptive statistics

Table 1 presents the descriptive statistics of all the variables in the regression analysis, including CVC variables, social trust variables, and corporate characteristic variables. Continuous variables are winsorized at the 1% and 99% levels to mitigate the impact of outliers. Table 1 reports the mean, standard deviation, minimum value, maximum value, and quantiles for each variable. During 2006–2018, CVC has a mean of 0.161 and a standard deviation of 0.368. One can notice that CVC occurred in 16.1% of the total observations, which implies that CVC of Chinese listed companies is relatively limited. The average of AMOUNT has a mean of 1.388 and a standard deviation of 3.245, and the value ranges from a minimum of 0 to a maximum of 11.510. It also reflects that many companies have not yet conducted CVC and the amount is not substantial. The maximum and minimum values of TRUST are 4.487 and 4.167 respectively, with a standard deviation of 0.086, indicating that the level of social trust varies by company location. The mean and median of TRUST are both around 4.2, indicating that the average social trust in China is at a medium-high level. Among different regions, social trust is higher in Beijing and Shanghai while relatively low in Heilongjiang and Guangxi. Then, the statistical data at the company level are also reported. The average TOP1 is about 0.358, with a standard deviation of 0.152. The IDR averages 0.371 with a standard deviation of 0.052. The average ROA is about 0.040, with a standard deviation of 0.060. The average AGE is about 15.290, with a standard deviation of 5.609. The average GROW is about 0.189, with a standard deviation of 0.473. The VC and SOE average 0.138 and 0.427, respectively. The average of SIZE is 21.933, and the firms are moderately leveraged with LEV of 0.439. Overall, the sufficient variation of key dependent variables, independent variables, and control variables provides confidence in the validity of the empirical research findings.

**Table 1:** Descriptive statistics.

VARIABLE	N	Mean	Sd	Min	Max	P25	P50	P75
CVC	27823	0.161	0.368	0	1	0	0	0
AMOUNT	27823	1.388	3.245	0	11.510	0	0	0
TRUST	27823	4.277	0.086	4.167	4.487	4.212	4.257	4.295
TOP1	27823	0.358	0.152	0.088	0.758	0.238	0.340	0.464
IDR	27823	0.371	0.052	0.300	0.571	0.333	0.333	0.400
VC	27823	0.138	0.345	0	1	0	0	0
ROA	27823	0.040	0.060	-0.243	0.205	0.015	0.039	0.069
LEV	27823	0.439	0.216	0.049	0.987	0.267	0.433	0.600
GROW	27823	0.189	0.473	-0.574	3.252	0	0.101	0.273
SIZE	27823	21.933	1.304	19.257	25.937	20.988	21.765	22.674
AGE	27823	15.290	5.609	3	29	11	15	19
SOE	27823	0.427	0.495	0	1	0	0	1

Notes: CVC and AMOUNT measure the number of deals and amount of CVC. TRUST is social trust measured by CEI index. TOP1 is value of proportion of the largest shareholder. IDR is independent director ratio. VC is a dummy variable which is coded 1 if the established company is supported by private venture capital funds, and 0 otherwise. ROA is the ratio of net profit divided by total assets. LEV is financial leverage. GROW is the growth rate of revenue from core business. SIZE is the natural logarithm of corporate assets. AGE is the natural logarithm of the company’s establishment time. SOE is a dummy variable which is coded 1 for State-owned firms and 0 otherwise.

### 4.2 Correlations

Table 2 reports the correlation between variables used in the base model. The results show that the correlation between TRUST and CVC is positive and significant (*ρ* = 0.042; at *p*<0.01), which provides preliminary support for a positive association between social trust and CVC. Moreover, the correlation between TRUST and AMOUNT is positive and significant (*ρ* = 0.047; at *p*<0.01), which is consistent with expectation. The correlations between other control variables are less than 0.5, indicating no multicollinearity in the model.

**Table 2:** Correlations.

	CVC	AMOUNT	TRUST	TOP1	IDR	VC	ROA	LEV	GROW	SIZE	AGE	SOE
CVC	1											
AMOUNT	0.975***	1										
TRUST	0.042***	0.047***	1									
TOP1	-0.055***	-0.049***	0.061***	1								
IDR	0.021***	0.022***	0.047***	0.040***	1							
VC	0.015**	0.011*	-0.011*	-0.088***	-0.023***	1						
ROA	0.022***	0.019***	0.025***	0.124***	-0.019***	0.056***	1					
LEV	-0.023***	-0.012*	-0.048***	0.043***	-0.024***	-0.136***	-0.394***	1				
GROW	0.074***	0.080***	-0.018***	0.013**	0.005	0.010*	0.164***	0.055***	1			
SIZE	0.069***	0.094***	0.135***	0.203***	0.018***	-0.127***	-0.008	0.403***	0.067***	1		
AGE	-0.022***	-0.013**	0.072***	-0.145***	0.012**	-0.071***	-0.104***	0.147***	0.002	0.191***	1	
SOE	-0.109***	-0.103***	0.009	0.203***	-0.082***	-0.143***	-0.127***	0.307***	-0.035***	0.313***	0.098***	1

**Fig 1:**
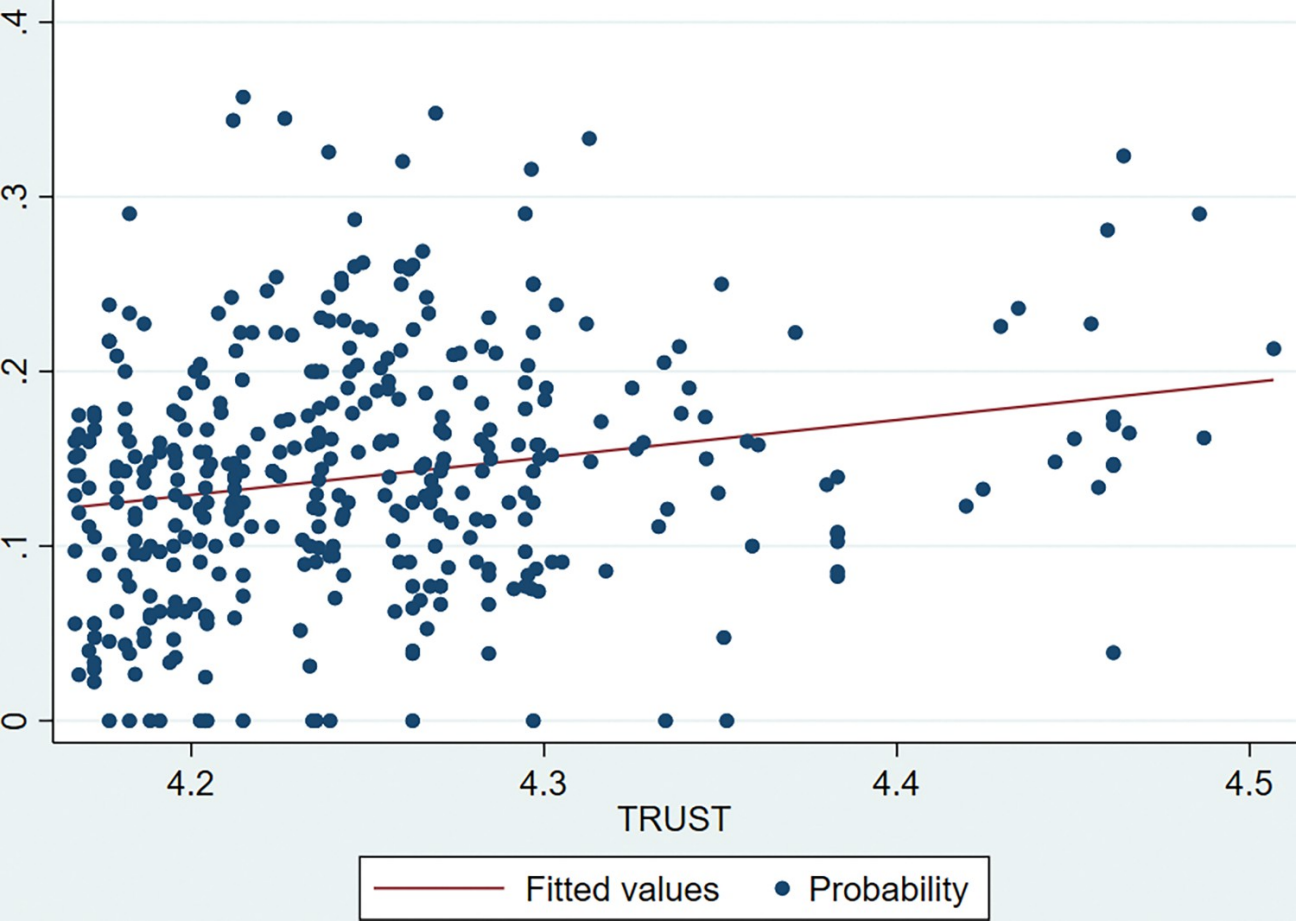
Trend graph between TRUST and CVC probability.

**Fig 2:**
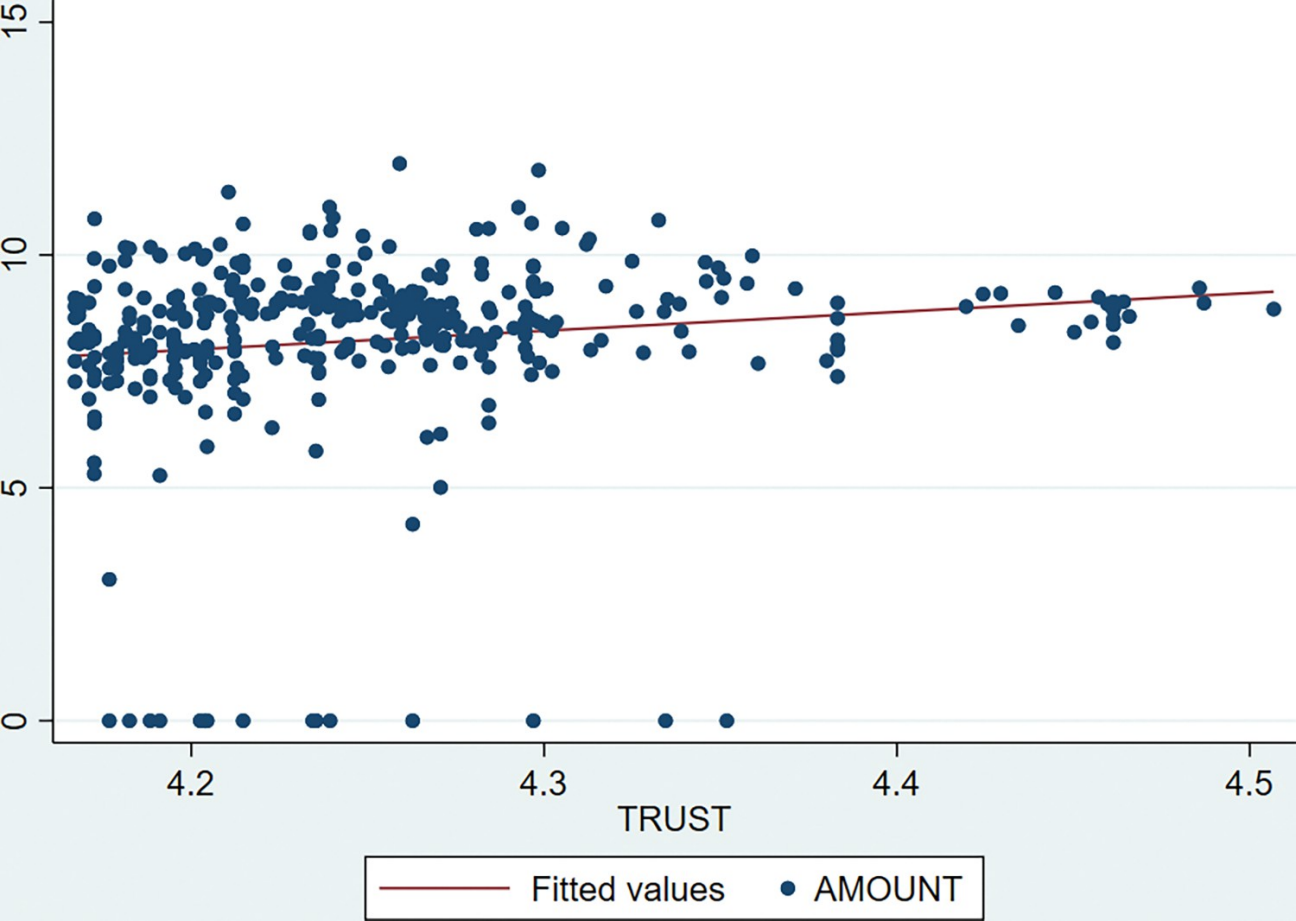
Trend graph between TRUST and CVC amount.

Figs 1 and 2 shows the scatter diagram of TRUST with the probability and the size of CVC. It can be visualized that both CVC probability and AMOUNT show a significant positive correlation with TRUST by fitted lines. That is, a higher level of social trust corresponds to positive CVC activities. The specific causal relationship between these two remains to be rigorously tested empirically.

### 4.3 Baseline regression results

Table 3 presents the baseline results of model (1). In both columns (1) and (4), the coefficient of TRUST is positive and significant (*β* = 1.272; at *p*<0.01; *β* = 1.760; at *p*<0.01, respectively), which clearly supports the hypothesis H1a and H1b that TRUST raises the likelihood and size of CVC. Adding the control variables that may affect CVC, columns (2) and (5) still show the positive effect of TRUST (*β* = 0.946; at *p*<0.01; *β* = 1.198; at *p*<0.01, respectively). Similar results (*β* = 0.596; at *p*<0.01; *β* = 0.817; at *p*<0.01, respectively) can still be observed when further controlling for year and industry in columns (3) and (6) of Table 3. The coefficients suggest that firms in the regions with higher social trust are more willing to engage in CVC deals. Hypotheses H1a and H1b are validated.

**Table 3:** Impact of social trust on corporate venture capital.

VARIABLES	Dependent variable = CVC			Dependent variable = AMOUNT		
	(1)	(2)	(3)	(4)	(5)	(6)
TRUST	1.272***	0.946***	0.596***	1.760***	1.198***	0.817***
	(7.131)	(4.949)	(2.821)	(7.558)	(5.132)	(3.265)
TOP1		-1.130***	-1.050***		-1.306***	-1.233***
		(-9.458)	(-8.587)		(-9.614)	(-9.018)
IDR		0.465	0.178		0.535	0.231
		(1.484)	(0.557)		(1.410)	(0.614)
VC		0.023	0.012		0.008	-0.009
		(0.476)	(0.250)		(0.134)	(-0.158)
ROA		-0.638**	-0.326		-1.091***	-0.639**
		(-2.142)	(-1.077)		(-3.377)	(-1.981)
LEV		-0.569***	-0.128		-0.590***	-0.069
		(-6.103)	(-1.284)		(-5.906)	(-0.659)
GROW		0.341***	0.357***		0.494***	0.514***
		(11.707)	(11.864)		(10.583)	(11.048)
SIZE		0.296***	0.271***		0.395***	0.354***
		(20.889)	(17.553)		(22.917)	(19.216)
AGE		-0.022***	-0.027***		-0.023***	-0.030***
		(-6.912)	(-7.064)		(-6.180)	(-6.801)
SOE		-0.718***	-0.702***		-0.813***	-0.775***
		(-17.990)	(-16.002)		(-19.407)	(-17.675)
Constant	-7.093***	-11.191***	-9.991***	-6.139***	-11.227***	-9.184***
	(-9.280)	(-13.687)	(-10.595)	(-6.176)	(-11.176)	(-8.192)
Year	N	N	Y	N	N	Y
Industry	N	N	Y	N	N	Y
Observations	27,823	27,823	27,823	27,823	27,823	27,823
R^2^ / Pseudo R^2^	0.056	0.060	0.057	0.002	0.039	0.058

Note

***, **, and * indicate 1%, 5%, and 10% significance, respectively.

### 4.4 Robustness tests

The baseline regression results may be confounded by indicator measurement errors and omitted variable bias. This study uses the methods of instrument variable and indicator replacement to perform robustness tests to enhance the reliability of the findings.

#### 4.4.1 Alternative measures of social trust

To pinpoint the social trust in the location of investors, the provincial-level index is replaced with the city-level (TRUST2). Meanwhile, following the study of Wu et al. [12], Chen et al. [75] and Qiu et al. [18], another measurement of social trust is introduced as an alternative, using TRUST_CESS as TRUST3. TRUST_CESS is derived from the *China Entrepreneurial Survey System* in 2000, which collected a survey from over 5,000 business leaders in all provinces in China [57]. Specifically, the index of TRUST_CESS is assessed by managers’ responses to the survey question *‘‘According to your experience*, *could you list the top five provinces where enterprises are most trustworthy*?*”* The results are shown in Table 4, which are consistent with the previous results and illustrate the stability of the original results.

**Table 4:** Robust regression of alternative social trust.

VARIABLES	Dependent variable = CVC		Dependent variable = AMOUNT	
	(1)	(2)	(3)	(4)
	TRUST = TRUST2	TRUST = TRUST3	TRUST = TRUST2	TRUST = TRUST3
TRUST	0.996***	0.001***	1.238***	0.001***
	(4.298)	(3.072)	(4.426)	(3.236)
TOP1	-1.043***	-1.053***	-1.259***	-1.233***
	(-8.281)	(-8.609)	(-8.613)	(-9.023)
IDR	0.259	0.171	0.326	0.224
	(0.792)	(0.534)	(0.817)	(0.594)
VC	0.009	0.011	-0.020	-0.013
	(0.179)	(0.222)	(-0.329)	(-0.218)
ROA	-0.311	-0.337	-0.635*	-0.657**
	(-0.992)	(-1.116)	(-1.828)	(-2.036)
LEV	-0.137	-0.121	-0.077	-0.061
	(-1.326)	(-1.209)	(-0.679)	(-0.578)
GROW	0.360***	0.358***	0.531***	0.515***
	(11.534)	(11.894)	(10.726)	(11.083)
SIZE	0.276***	0.272***	0.371***	0.355***
	(17.225)	(17.540)	(18.806)	(19.180)
AGE	-0.024***	-0.027***	-0.028***	-0.031***
	(-6.300)	(-7.108)	(-6.013)	(-6.896)
SOE	-0.722***	-0.689***	-0.824***	-0.759***
	(-15.848)	(-15.706)	(-17.427)	(-17.277)
Constant	-11.216***	-7.526***	-11.048***	-5.793***
	(-10.779)	(-19.851)	(-8.747)	(-13.622)
Year	Y	Y	Y	Y
Industry	Y	Y	Y	Y
Observations	25,207	27,823	25,207	27,823
R^2^ / Pseudo R^2^	0.056	0.059	0.057	0.058

Note

***, **, and * indicate 1%, 5%, and 10% significance, respectively.

#### 4.4.2 Alternative measures of corporate venture capital

The number of CVC deals (TIME) is considered as a surrogate variable for CVC decision-making. Also, the total investment is replaced with AMOUNT2, referring to the largest amount of the CVC deal. In addition, to eliminate the impact of firm size on investment, the relative number between CVC and the capital stock serves as AMOUNT3 to represent the size of CVC. As expected, in Table 5, TRUST is significantly positively correlated with TIME, AMOUNT2 and AMOUNT3. The overall results are similar, strengthening the stability of the conclusions of this paper.

**Table 5:** Robust regression of alternative corporate venture capital.

VARIABLES	Dependent variable = TIME	Dependent variable = AMOUNT2	Dependent variable = AMOUNT3
	(1)	(2)	(3)
TRUST	0.130**	0.802***	0.054***
	(2.207)	(3.246)	(2.937)
TOP1	-0.241***	-1.217***	-0.027***
	(-7.661)	(-9.026)	(-3.523)
IDR	0.176	0.216	0.097***
	(1.112)	(0.580)	(4.017)
VC	-0.004	-0.011	-0.002
	(-0.357)	(-0.187)	(-0.610)
ROA	-0.032	-0.636**	-0.084**
	(-0.488)	(-1.996)	(-2.474)
LEV	0.027	-0.074	-0.023**
	(0.995)	(-0.712)	(-1.989)
GROW	0.103***	0.505***	0.021***
	(9.668)	(11.008)	(7.457)
SIZE	0.069***	0.349***	-0.022***
	(14.560)	(19.155)	(-11.751)
AGE	-0.006***	-0.030***	-0.001***
	(-5.998)	(-6.757)	(-4.819)
SOE	-0.154***	-0.764***	-0.027***
	(-13.236)	(-17.635)	(-9.537)
Constant	-1.756***	-9.015***	0.292***
	(-7.215)	(-8.146)	(4.133)
Year	Y	Y	Y
Industry	Y	Y	Y
Observations	27,823	27,823	27,823
R^2^ / Pseudo R^2^	0.050	0.058	0.049

Note

***, **, and * indicate 1%, 5%, and 10% significance, respectively.

#### 4.4.3 Cross-period analysis

Given the enduring and extensive influence of social trust, the analysis incorporates CVC for both one-period and two-period forecasts as the dependent variable. This approach is to scrutinize the delayed effects of social trust on corporate venture capital, facilitating a temporal analysis. Table 6 shows that the significance of TRUST remains consistent with the main findings in the previous section.

**Table 6:** Robust regression of CVC for future one-period and two-period.

VARIABLES	Dependent variable = CVC		Dependent variable = AMOUNT	
	(1)	(2)	(3)	(4)
	F1. CVC	F2. CVC	F1. AMOUNT	F2. AMOUNT
TRUST	0.465**	0.492**	0.730***	0.790***
	(2.092)	(1.999)	(2.646)	(2.610)
TOP1	-0.616***	-0.357***	-0.779***	-0.467***
	(-4.970)	(-2.724)	(-5.096)	(-2.809)
IDR	0.376	0.527	0.527	0.831*
	(1.152)	(1.510)	(1.265)	(1.817)
VC	0.190***	0.179***	0.216***	0.226***
	(3.898)	(3.422)	(3.206)	(2.999)
ROA	1.350***	1.499***	1.122***	1.441***
	(3.996)	(4.140)	(2.953)	(3.460)
LEV	-0.317***	-0.067	-0.332***	-0.039
	(-3.012)	(-0.613)	(-2.727)	(-0.296)
GROW	0.259***	0.235***	0.379***	0.348***
	(8.030)	(6.717)	(7.693)	(6.417)
SIZE	0.178***	0.091***	0.251***	0.145***
	(10.785)	(5.017)	(12.141)	(6.390)
AGE	-0.029***	-0.029***	-0.035***	-0.037***
	(-7.258)	(-6.888)	(-7.039)	(-6.635)
SOE	-0.691***	-0.724***	-0.816***	-0.876***
	(-15.552)	(-15.562)	(-16.878)	(-16.781)
Constant	-6.991***	-5.130***	-6.413***	-4.468***
	(-7.048)	(-4.675)	(-5.184)	(-3.290)
Year	Y	Y	Y	Y
Industry	Y	Y	Y	Y
Observations	24,419	21,230	24,419	21,237
R^2^ / Pseudo R^2^	0.052	0.051	0.051	0.050

Note

***, **, and * indicate 1%, 5%, and 10% significance, respectively.

#### 4.4.4 Robust regression of time subinterval

With the high external risks and uncertainties caused by the global financial crisis in 2008, the enthusiasm of enterprises for venture capital has been significantly affected. The sample period is set to begin in 2010 or 2011. The results are shown in Table 7. The coefficient of TRUST is still significant, which is qualitatively similar to the earlier finding.

**Table 7:** Robust regression of time subinterval.

VARIABLES	Dependent variable = CVC		Dependent variable = AMOUNT	
	(1)	(2)	(3)	(4)
	2010–2018	2011–2018	2010–2018	2011–2018
TRUST	0.648***	0.731***	0.938***	1.030***
	(2.824)	(3.062)	(3.180)	(3.267)
TOP1	-1.010***	-0.908***	-1.282***	-1.201***
	(-7.679)	(-6.685)	(-8.134)	(-7.178)
IDR	0.353	0.506	0.428	0.618
	(1.043)	(1.448)	(1.010)	(1.378)
VC	0.019	0.029	-0.015	0.001
	(0.372)	(0.550)	(-0.229)	(0.010)
ROA	-0.708**	-0.817**	-1.110***	-1.254***
	(-2.137)	(-2.394)	(-2.784)	(-2.963)
LEV	-0.154	-0.162	-0.108	-0.141
	(-1.407)	(-1.404)	(-0.851)	(-1.025)
GROW	0.369***	0.361***	0.569***	0.571***
	(11.213)	(10.583)	(10.345)	(9.802)
SIZE	0.260***	0.251***	0.365***	0.362***
	(15.239)	(14.089)	(16.987)	(15.830)
AGE	-0.026***	-0.025***	-0.030***	-0.030***
	(-6.464)	(-6.038)	(-6.091)	(-5.703)
SOE	-0.744***	-0.782***	-0.882***	-0.933***
	(-15.188)	(-15.241)	(-16.935)	(-16.858)
Constant	-9.054***	-9.224***	-9.359***	-9.578***
	(-8.809)	(-8.604)	(-7.059)	(-6.768)
Year	Y	Y	Y	Y
Industry	Y	Y	Y	Y
Observations	22,495	20,668	22,495	20,668
R^2^ / Pseudo R^2^	0.054	0.054	0.055	0.055

Note

***, **, and * indicate 1%, 5%, and 10% significance, respectively.

#### 4.4.5 Additional control variables

Only governance, financial and organizational attributes are initially considered among the control variables, but regional macroeconomic conditions may also have an impact on CVC. To mitigate concern about omitted bias, this paper adds the economic development of the firm’s location GDPCAP [76] and the regional financial development FD in the baseline model. GDPCAP is GDP per capita growth from the *China Statistical Yearbook*. FD is the level of marketization of the financial sector by province from the *Marketization Index of China’s Provinces*: *NERI Report 2018*. The results are shown in columns (1) and (2) of Table 8. The significance of TRUST does not fluctuate compared to the baseline regression.

**Table 8:** Robust regression of additional control variables.

VARIABLES	Dependent variable = CVC	Dependent variable = AMOUNT
	(1)	(2)
TRUST	0.747***	0.987***
	(3.428)	(3.744)
TOP1	-1.053***	-1.229***
	(-8.595)	(-8.992)
IDR	0.179	0.227
	(0.559)	(0.602)
VC	0.015	-0.008
	(0.299)	(-0.135)
ROA	-0.378	-0.678**
	(-1.246)	(-2.098)
LEV	-0.138	-0.079
	(-1.381)	(-0.751)
GROW	0.358***	0.513***
	(11.857)	(11.011)
SIZE	0.274***	0.356***
	(17.679)	(19.320)
AGE	-0.027***	-0.030***
	(-7.099)	(-6.812)
SOE	-0.691***	-0.770***
	(-15.428)	(-17.032)
GDPCAP	1.663***	1.664**
	(2.728)	(2.522)
FD	0.019**	0.013
	(1.976)	(1.176)
Constant	-11.048***	-10.264***
	(-10.979)	(-8.445)
Year	Y	Y
Industry	Y	Y
Observations	27,823	27,823
R^2^ / Pseudo R^2^	0.060	0.059

Note

***, **, and * indicate 1%, 5%, and 10% significance, respectively.

#### 4.4.6 Endogeneity issue

Although potential errors have been attenuated in the above tests, there are still concerns about endogeneity issues. Referring to Guiso et al. [11], the per capita voluntary blood donation rate (BLOOD) in each province is used as an instrument of trust to solve the endogeneity problem. The voluntary blood donation rate reflects the social morality of a region [11], and social morality usually means trust between human beings. Therefore, the instrumental variable is correlated with social trust. In addition, there is no direct correlation between the BLOOD and CVC, meaning the instrument is qualified. The result of two‐stage least squares regression is shown in Table 9. The first-stage regression results illustrate that the coefficient on instrumented BLOOD and TRUST is significant, indicating a positive correlation between these two variables. The two-stage regression results showed that TRUST still has a significant positive relationship with CVC. When using the instrumental variable, the association between social trust and CVC is robust in controlling for the endogeneity issue.

**Table 9:** Two-stage regression results.

VARIABLES	Dependent variable = TRUST	Dependent variable = CVC	Dependent variable = AMOUNT
	(1)	(2)	(3)
BLOOD	0.212***		
	(275.492)		
TRUST		0.499***	1.198***
		(3.606)	(4.050)
TOP1	-0.000	-0.592***	-1.243***
	(-0.279)	(-8.847)	(-9.099)
IDR	-0.007	0.090	0.224
	(-1.445)	(0.505)	(0.595)
VC	-0.003***	0.012	-0.008
	(-3.877)	(0.428)	(-0.137)
ROA	0.003	-0.188	-0.637**
	(0.594)	(-1.016)	(-1.974)
LEV	0.004**	-0.065	-0.055
	(2.493)	(-1.094)	(-0.525)
GROW	0.000	0.210***	0.515***
	(0.459)	(11.183)	(11.066)
SIZE	0.001***	0.151***	0.352***
	(3.216)	(16.204)	(19.023)
AGE	0.000	-0.014***	-0.030***
	(0.559)	(-7.205)	(-6.757)
SOE	0.010***	-0.383***	-0.778***
	(17.263)	(-16.432)	(-17.753)
Constant	4.011***	-6.258***	-10.743***
	(739.394)	(-10.379)	(-8.332)
YEAR	Y	Y	Y
INDUSTRY	Y	Y	Y
Observations	27,823	27,823	27,823
R^2^ / Pseudo R^2^	0.782	-	0.058

Note

***, **, and * indicate 1%, 5%, and 10% significance, respectively.

### 4.5 Moderation effect

This paper considers the moderating effects of external and internal factors, including state ownership, CEO duality, marketization level and legal environment. The regression results are shown in Tables 10–13.

**Table 10:** Effect of SOE on the relation between social trust and corporate venture capital.

VARIABLES	Dependent variable = CVC		Dependent variable = AMOUNT	
	(1)	(2)	(3)	(4)
	SOE	NSOE	SOE	NSOE
TRUST	0.369	0.877***	0.438	1.313***
	(1.104)	(3.161)	(1.414)	(3.299)
TOP1	-1.455***	-0.668***	-1.187***	-0.877***
	(-6.512)	(-4.472)	(-6.064)	(-4.566)
IDR	-0.517	0.713*	-0.288	0.951*
	(-0.882)	(1.810)	(-0.562)	(1.796)
VC	0.268***	-0.045	0.260**	-0.068
	(2.703)	(-0.800)	(2.565)	(-0.976)
ROA	1.704***	-1.250***	0.927**	-1.719***
	(2.858)	(-3.515)	(2.057)	(-3.919)
LEV	0.015	-0.230*	-0.040	-0.104
	(0.085)	(-1.847)	(-0.274)	(-0.705)
GROW	0.305***	0.369***	0.337***	0.581***
	(5.973)	(9.552)	(5.187)	(9.024)
SIZE	0.238***	0.323***	0.261***	0.475***
	(9.332)	(15.321)	(10.497)	(16.796)
AGE	-0.010	-0.033***	-0.006	-0.040***
	(-1.279)	(-7.373)	(-0.797)	(-7.051)
Constant	-8.439***	-12.777***	-6.029***	-14.626***
	(-5.768)	(-9.906)	(-4.303)	(-8.077)
Year	Y	Y	Y	Y
Industry	Y	Y	Y	Y
Observations	11,870	15,943	11,880	15,943
R^2^ / Pseudo R^2^	0.047	0.056	0.038	0.066

Note

***, **, and * indicate 1%, 5%, and 10% significance, respectively.

**Table 11:** Effect of CEO duality on the relation between social trust and corporate venture capital.

VARIABLES	Dependent variable = CVC		Dependent variable = AMOUNT	
	(1)	(2)	(3)	(4)
	DUAL = 1	DUAL = 0	DUAL = 1	DUAL = 0
TRUST	0.564	0.593**	0.662	0.810***
	(1.345)	(2.395)	(1.205)	(2.889)
TOP1	-0.634***	-1.174***	-0.994***	-1.252***
	(-2.713)	(-8.067)	(-3.379)	(-8.067)
IDR	0.189	0.001	0.717	-0.140
	(0.320)	(0.003)	(0.932)	(-0.325)
VC	-0.100	0.073	-0.157	0.068
	(-1.188)	(1.204)	(-1.525)	(0.967)
ROA	-1.799***	0.170	-2.424***	-0.168
	(-3.185)	(0.466)	(-3.591)	(-0.453)
LEV	-0.490**	-0.024	-0.385*	-0.016
	(-2.478)	(-0.201)	(-1.699)	(-0.136)
GROW	0.463***	0.326***	0.729***	0.450***
	(6.764)	(9.522)	(6.490)	(8.858)
SIZE	0.323***	0.267***	0.452***	0.339***
	(9.918)	(14.814)	(10.845)	(16.254)
AGE	-0.042***	-0.020***	-0.056***	-0.020***
	(-5.792)	(-4.463)	(-6.295)	(-3.850)
SOE	-0.658***	-0.668***	-0.770***	-0.726***
	(-5.453)	(-13.786)	(-6.910)	(-14.841)
Constant	-11.492***	-9.757***	-11.292***	-8.676***
	(-5.794)	(-8.949)	(-4.475)	(-6.948)
Year	Y	Y	Y	Y
Industry	Y	Y	Y	Y
Observations	6,777	21,046	6,777	21,046
R^2^ / Pseudo R^2^	0.074	0.056	0.084	0.053

Note

***, **, and * indicate 1%, 5%, and 10% significance, respectively.

**Table 12:** Effect of MARKET on the relation between social trust and corporate venture capital.

VARIABLES	Dependent variable = CVC		Dependent variable = AMOUNT	
	(1)	(2)	(3)	(4)
	MARKET = 1	MARKET = 0	MARKET = 1	MARKET = 0
TRUST	0.662***	-1.431	0.913***	-1.201
	(2.842)	(-1.061)	(3.325)	(-0.900)
TOP1	-0.948***	-1.213***	-1.153***	-1.308***
	(-6.708)	(-4.896)	(-7.218)	(-4.909)
IDR	0.162	0.108	0.284	-0.027
	(0.442)	(0.161)	(0.643)	(-0.037)
VC	-0.030	0.119	-0.063	0.119
	(-0.534)	(1.221)	(-0.935)	(1.060)
ROA	-0.758**	0.635	-1.040***	0.090
	(-2.151)	(1.036)	(-2.668)	(0.156)
LEV	-0.186	-0.062	-0.110	-0.067
	(-1.576)	(-0.315)	(-0.864)	(-0.354)
GROW	0.396***	0.262***	0.578***	0.356***
	(10.858)	(4.661)	(10.127)	(4.374)
SIZE	0.260***	0.334***	0.342***	0.405***
	(14.082)	(11.074)	(15.507)	(11.862)
AGE	-0.027***	-0.023***	-0.032***	-0.021**
	(-6.200)	(-2.828)	(-6.227)	(-2.372)
SOE	-0.736***	-0.615***	-0.801***	-0.678***
	(-13.396)	(-7.794)	(-14.832)	(-8.526)
Constant	-11.165***	-2.229	-10.254***	-1.066
	(-10.201)	(-0.392)	(-8.144)	(-0.188)
Year	Y	Y	Y	Y
Industry	Y	Y	Y	Y
Observations	20,127	7,693	20,127	7,696
R^2^ / Pseudo R^2^	0.065	0.058	0.064	0.054

Note

***, **, and * indicate 1%, 5%, and 10% significance, respectively.

**Table 13:** Effect of LAW on the relation between social trust and corporate venture capital.

VARIABLES	Dependent variable = CVC		Dependent variable = AMOUNT	
	(1)	(2)	(3)	(4)
	LAW = 1	LAW = 0	LAW = 1	LAW = 0
TRUST	0.643***	-0.191	0.886***	0.319
	(2.706)	(-0.146)	(3.145)	(0.251)
TOP1	-0.870***	-1.463***	-1.072***	-1.522***
	(-6.072)	(-6.130)	(-6.536)	(-6.067)
IDR	0.320	-0.397	0.459	-0.461
	(0.852)	(-0.632)	(0.997)	(-0.704)
VC	-0.045	0.169*	-0.083	0.176
	(-0.794)	(1.773)	(-1.216)	(1.625)
ROA	-0.788**	0.550	-1.062**	-0.026
	(-2.167)	(0.989)	(-2.566)	(-0.051)
LEV	-0.181	-0.074	-0.119	-0.060
	(-1.511)	(-0.401)	(-0.902)	(-0.346)
GROW	0.410***	0.263***	0.600***	0.353***
	(10.805)	(5.042)	(10.056)	(4.748)
SIZE	0.256***	0.329***	0.341***	0.399***
	(13.673)	(11.320)	(14.987)	(12.377)
AGE	-0.028***	-0.018**	-0.033***	-0.016*
	(-6.320)	(-2.404)	(-6.346)	(-1.858)
SOE	-0.777***	-0.501***	-0.848***	-0.562***
	(-13.864)	(-6.561)	(-15.404)	(-7.316)
Constant	-10.333***	-7.466	-9.635***	-7.585
	(-9.394)	(-1.356)	(-7.423)	(-1.402)
Year	Y	Y	Y	Y
Industry	Y	Y	Y	Y
Observations	19,401	8,420	19,401	8,422
R^2^ / Pseudo R^2^	0.063	0.057	0.063	0.055

Note

***, **, and * indicate 1%, 5%, and 10% significance, respectively.

Table 10 reports the group test results on the state ownership. The coefficients of TRUST in the state-owned enterprise group are not significant. Among non-state-owned enterprises, the coefficients of TRUST are positive and significant (*β* = 0.877; at *p*<0.01; *β* = 1.313, at *p*<0.01, respectively). The result fully demonstrates a difference in which social trust works in SOEs and non-SOEs. Compared with state-owned enterprises, social trust has a more significant role in promoting CVC in non-state-owned enterprises. Table 11 reports the regression results with the division by CEO duality. For companies whose CEO and chairman are not the same person, the coefficient of TRUST on CVC is positive and significant (*β* = 0.593; at *p*<0.05), so as the coefficient on AMOUNT (*β* = 0.810; at *p*<0.01). The results support hypothesis H2b that the separation of the two roles can strengthen the promotion effect of TRUST on CVC.

Table 12 reveals that the high degree of marketization could help enhance the positive impact of social trust on CVC (*β* = 0.662; at *p*<0.01; *β* = 0.913, at *p*<0.01, respectively). As expected, hypothesis H2c is verified. Table 13 indicates that social trust and the legal environment are not mutually alternative but complementary. Hypothesis H2d is not validated; this may be since social trust is often affected and protected by the sound legal environment [10]. The existence of law builds a bridge for the trust of trading parties, and reduces the potential moral hazard of cost and information asymmetry caused by distrust. Law is the necessary foundation for the effect of social trust, which expands the radius of social trust and builds an excellent ecological environment for corporate venture capital.

### 4.6 Social trust and CVC effect

In this section, the research focus is on established companies with CVC deals. It is questioned what CVC value could be created by social trust. According to the previous assumptions, social trust helps both parties establish mutual expectations of reliability, facilitates information sharing, knowledge transfer and resource integration in the CVC deal, all of which may affect the performance and returns of the established companies. When companies carry out a CVC deal, the expected effects include financial returns and strategic benefits [77,78]. EBITDA and PATENT are set as indicators to test investment effects [79,80].

Considering that the effect may not be immediately visible, the time lag variables are set. Columns (1) and (2) in Table 14 indicate that the coefficients of TRUST are not significant in the regressions of financial return EBITDA in periods T, T+1. The coefficient of EBITDA for period T+2 is significant at the 10% level, illustrating the feedback of finance from social trust is a long-lasting process. Columns (4), (5) and (6) reveal significant correlations between TRUST and PATENT, suggesting the positive impact of social trust on CVC innovation outcomes. The higher the level of social trust, the more innovations the CVC creates. This section concludes that the improvement of CVC on financial performance is not visible for the time being, but the impact on innovation output has already appeared. This result also confirms the innovative value brought by trust-driven CVC [80]. Hypothesis H3 is validated.

**Table 14:** Social trust and CVC effect.

VARIABLES	Dependent variable = EBITDA			Dependent variable = PATENT		
	(1)	(2)	(3)	(4)	(5)	(6)
	T	T+1	T+2	T	T+1	T+2
TRUST	0.097	0.016	0.273*	3.174***	2.931***	3.428***
	(1.367)	(0.138)	(1.893)	(5.197)	(5.002)	(5.480)
TOP1	0.082*	0.224***	0.304***	-0.688*	-0.388	-0.106
	(1.823)	(3.660)	(3.910)	(-1.801)	(-1.112)	(-0.293)
IDR	-0.094	-0.121	-0.191	2.204**	1.854*	1.309
	(-0.797)	(-0.743)	(-0.895)	(2.125)	(1.793)	(1.217)
VC	-0.015	-0.049*	-0.012	0.283**	0.107	0.290*
	(-1.070)	(-1.935)	(-0.353)	(1.968)	(0.770)	(1.838)
ROA	13.113***	6.142***	5.619***	1.637	4.433***	4.899***
	(51.654)	(18.550)	(14.682)	(1.088)	(3.006)	(3.265)
LEV	0.505***	0.280***	0.203**	-0.418	-0.655	-0.472
	(9.681)	(3.929)	(2.147)	(-1.017)	(-1.634)	(-1.125)
GROW	-0.024*	0.070***	0.054**	-0.176	-0.053	-0.018
	(-1.670)	(4.035)	(2.393)	(-1.144)	(-0.359)	(-0.127)
SIZE	1.013***	0.977***	0.959***	1.168***	1.080***	1.030***
	(121.297)	(87.095)	(68.436)	(19.193)	(18.498)	(16.192)
AGE	0.001	-0.002	-0.003	-0.018*	-0.028***	-0.032***
	(0.794)	(-1.241)	(-1.468)	(-1.699)	(-2.701)	(-2.832)
SOE	0.024	-0.047*	-0.085***	-0.019	0.285**	0.340**
	(1.401)	(-1.945)	(-2.873)	(-0.144)	(2.322)	(2.416)
Constant	-4.052***	-2.206***	-3.034***	-40.918***	-38.098***	-38.283***
	(-11.810)	(-4.129)	(-4.779)	(-16.265)	(-15.667)	(-15.349)
Year	Y	Y	Y	Y	Y	Y
Industry	Y	Y	Y	Y	Y	Y
Observations	4,326	4,245	4,148	4,491	4,490	4,003
R-squared	0.917	0.829	0.740	0.120	0.119	0.120

Note

***, **, and * indicate 1%, 5%, and 10% significance, respectively.

### 4.7 Additional analyses

Additional investigations are conducted to further understand the potential path by which social trust influences the decision of corporate venture capital. Williamson [81] and Bottazzi et al. [21] believe that social trust enhances the confidence and expectations of enterprises for the future, making them risk-competent. This view is indeed supported by data. A test of social trust on the ability of corporate risk-taking is performed. Following the approach of John et al. [82] and Faccio et al. [83], the volatility of industry-adjusted profitability is used to measure corporate risk-taking. As shown in Eqs (4)–(6), the rolling calculation of industry-adjusted standard deviation and extreme deviation of ROA are considered as RISKTAKE1 and RISKTAKE2. In Table 15, significant positive relationships between social trust and corporate risk-taking are evidenced for either measure.


$$RISKTAKE1=\sqrt{\frac{1}{T-1}\overset{T}{\underset{t=1}{\sum }}{\left(Adj\_ROA_{i,t}-\frac{1}{T}Adj_{-}ROA_{i,t}\right)}^{2}}|T=3$$ (4)

Where

$$Adj_{-}ROA_{i,t}=\frac{EBIT_{i,t}}{ASSET_{i,t}}-\frac{1}{X}\overset{X}{\underset{k=1}{\sum }}\frac{EBIT_{i,t}}{ASSET_{i,t}}$$ (5)

$$RISKTAKE2=Max(Adj\_ROA_{i,t})-Min(Adj_{-}ROA_{i,t})$$ (6)

**Table 15:** Additional analyses.

VARIABLES	Dependent variable = RISKTAKE		Dependent variable = SYN	
	(1)	(2)	(3)	(4)
	RISKTAKE1	RISKTAKE2	SYN1	SYN2
TRUST	0.006***	0.012***	-0.330***	-0.264***
	(2.722)	(2.721)	(-4.397)	(-4.111)
TOP1	0.003*	0.005*	-0.189***	-0.222***
	(1.838)	(1.935)	(-4.485)	(-6.222)
IDR	0.020***	0.038***	-0.237**	0.004
	(5.416)	(5.368)	(-2.040)	(0.045)
VC	0.000	0.000	-0.071***	-0.061***
	(0.304)	(0.258)	(-3.885)	(-3.881)
ROA	-0.115***	-0.214***	-1.077***	-0.438***
	(-15.899)	(-15.965)	(-8.254)	(-4.038)
LEV	0.002	0.005	-0.371***	-0.448***
	(1.166)	(1.317)	(-9.304)	(-13.194)
GROW	0.005***	0.009***	-0.180***	-0.165***
	(7.542)	(7.489)	(-12.745)	(-13.627)
SIZE	-0.006***	-0.012***	0.155***	0.174***
	(-25.997)	(-26.170)	(23.977)	(31.682)
AGE	0.000***	0.000***	0.003**	-0.002**
	(5.477)	(5.575)	(2.452)	(-2.020)
SOE	-0.004***	-0.008***	0.178***	0.187***
	(-8.407)	(-8.388)	(13.222)	(16.007)
Constant	0.141***	0.267***	-2.596***	-2.753***
	(13.409)	(13.545)	(-7.792)	(-9.628)
Year	Y	Y	Y	Y
Industry	Y	Y	Y	Y
Observations	26,998	26,998	24,729	24,729
R^2^ / Pseudo R^2^	0.153	0.155	0.364	0.295

Note

***, **, and * indicate 1%, 5%, and 10% significance, respectively.

Here, *i* represents the firm. *T* indexes the year in the observation period, taking values from 1 to 3. *X* represents the total number of firms in the same industry. *EBIT* is earnings before interest and tax, and *ASSET* is total corporate assets.

Social trust positively contributes to reducing information friction by providing investors with more specific information in the transaction [36,43]. This study attempts to test the effect of social trust on asymmetric information. Kelly [84] argues that low R^2^ implies lower transmission efficiency, poorer quality of the information environment and greater information asymmetry. Referring to Morck et al. [85] and Kong et al. [86], stock price synchronicity is used to measure asymmetry information. The calculations are shown in Eqs (7)–(9). Columns (3) and (4) of Table 15 show that social trust decreases stock price synchronicity and reduces informational frictions in trading. A significant negative relationship is noted between TRUST and SYN, which is consistent with the findings of Qiu et al. [18]. These results further support our interpretation in the hypotheses.


$$R_{i,t}=\beta_{0}+\beta_{1}R_{m,t}+\varepsilon$$ (7)

$$R_{i,t}=\beta_{0}+\beta_{1}R_{m,t}+\beta_{2}R_{l,t}+\varepsilon$$ (8)

$$SYN=Ln(\frac{R_{i}^{2}}{1-R_{i}^{2}})$$ (9)

Here, R_*i*,*t*_ is the return of firm *i* in week *t*, R_*m*,*t*_ is the market return in week *t*, R_*l*,*t*_ is the industry return in week *t*. R^2^ represents the coefficient of determination from the estimation of Eqs (7) and (8). Since R^2^ could not meet the regression requirements of least squares, a logarithmic transformation of R^2^ is performed in Eq (9) [87].

## 5 Conclusion and discussion

### 5.1 Conclusion

Using data of listed companies in China from 2006–2018, this paper empirically examines the impact of social trust on CVC investment and CVC effects. The finding complements that of Bottazzi et al. [21], providing evidence that social trust could promote corporate venture capital. It is found that the higher the level of social trust in a region, the more favorable it is to motivate companies to carry out CVC. Meanwhile, the function of social trust is influenced by many factors. Heterogeneity demonstrates that the impact of social trust in promoting CVC is weakened by state ownership and the unity of CEO and chairman; however, it can be strengthened in environments with a high degree of marketization and the rule of law. The function of social trust depends on a stable economic background and favorable policy support to a certain extent. Additionally, higher social trust creates great CVC effects. In regions with higher social trust, the honesty and trustworthiness of both parties to a transaction reduce uncertainty, ultimately promoting efficient cooperative behavior and increasing the value created by CVC.

### 5.2 Discussion

Corporate venture capital is risky decision-making full of uncertainty, and it might be challenging to achieve the ideal transaction by endogenous contractual arrangements. It requires a suitable external mechanism or a soft environment for the growth of corporate venture capital. The conclusions confirm that a great social trust environment can significantly enhance corporate venture capital, which serves as a reminder of the urgent need for informal institutions’ vital role in corporate venture capital.

A higher level of support and assistance is required to create a trusting environment. Building a good business trust environment, enhancing inter-firm contracts and trust, and guiding firms to form long-term commitments are feasible strategies to motivate CVC. On the other hand, the results demonstrate that the promotion of social trust for CVC is more potent in regions with a well-established external environment. Formal and informal institutions have a synergistic multiplier effect for stimulating CVC. In order to sustain a fair and transparent formal institutional environment, the continuity and stability of formal regulatory policies are essential. Regulators must prioritize the integration development of both informal and formal institutions, construct complementary mechanisms and foster a conducive atmosphere for CVC.

### 5.3 Limitations and future research

Social trust is a relatively abstract concept that is often difficult to observe. Due to the lack of year-by-year data on the business credit environment, immediate changes in social trust are difficult to portray accurately. The data from questionnaire might be disturbed by factors such as respondents’ subjective emotions. In future studies, the reliability of research findings can be further improved if more appropriate social trust metrics can be constructed. Recent years have seen a rise in emerging economies, and the role of social trust as a soft constraint in these nations requires additional attention. This study only takes China, a typical representative of developing countries, as the research object. More countries from emerging markets could be included in future research, giving the findings deeper significance and wider application.
